# A Rare Case Report of Tuberculous Infection in a Previously Neglected Dysplastic Hip of a Young Woman

**DOI:** 10.5435/JAAOSGlobal-D-19-00144

**Published:** 2020-02-05

**Authors:** Sujata Aiyer, Aditya Raj, Swapneel Shah, Nandan Marathe, B.S. Sujith

**Affiliations:** From the Department Of Orthopedics, Seth G.S Medical College & King Edward Memorial Hospital, Parel (Dr. Aiyer, Dr. Raj, Dr. Shah, and Dr. Marathe); and the Department Of Orthopedics, T.N. Medical College (Dr. Sujith), Mumbai, India.

## Abstract

A 14-year-old girl presented with an insidious onset of left hip pain, limp, and intermittent fever for a 3-month duration. Patient had a history of toe walking since childhood which continued into adolescence. On radiographic investigations, she was found to have a dysplastic hip with fluid collection around the hip which was surgically drained. The microbiological investigations proved the presence of Mycobacterium tuberculosis (TB). Accordingly, she was started on anti-TB chemotherapy as per drug sensitivity. TB infection in a previously neglected dysplastic hip is not reported as per our knowledge and poses unique diagnostic and management difficulties.

Tuberculosis (TB) of the hip is the second most frequent site of occurrence after spine in osteoarticular TB. Many patients get diagnosed in the advanced stage of the disease owing to atypical clinical presentations despite the presence of an extended spectrum of modalities. In most cases of TB of the hip, there is a dilemma of accurate diagnosis owing to its presenting complaints of painful and restricted movements of the hip because several pathologies may mimic this presentation. In the pediatric age group, diseases such as juvenile idiopathic arthritis, Perthes disease, and pyogenic arthritis are differentials. Largely based on clinical suspicion, multiple radiological and microbiological investigations help in the early diagnosis of TB of the hip. With late presentations and diagnoses, the extent of the disease becomes increasingly severe with profound destruction of the joint. We present a case of a 14-year-old girl with TB of hip in a pre-existing unstable hip secondary to a neglected dysplastic hip.

Statement of informed consent: The patient was informed that the data including clinical photos concerning the case would be submitted for publication, and a written consent was taken accordingly.

## Case Report

**Figure 1:**
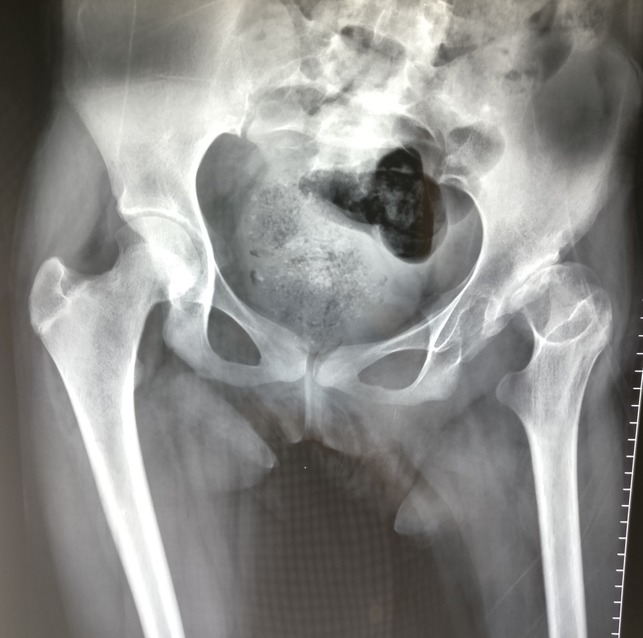
Radiograph showing the anterior-posterior view of the pelvis with both hips demonstrating a neglected dysplastic hip on the left side.

**Figure 2:**
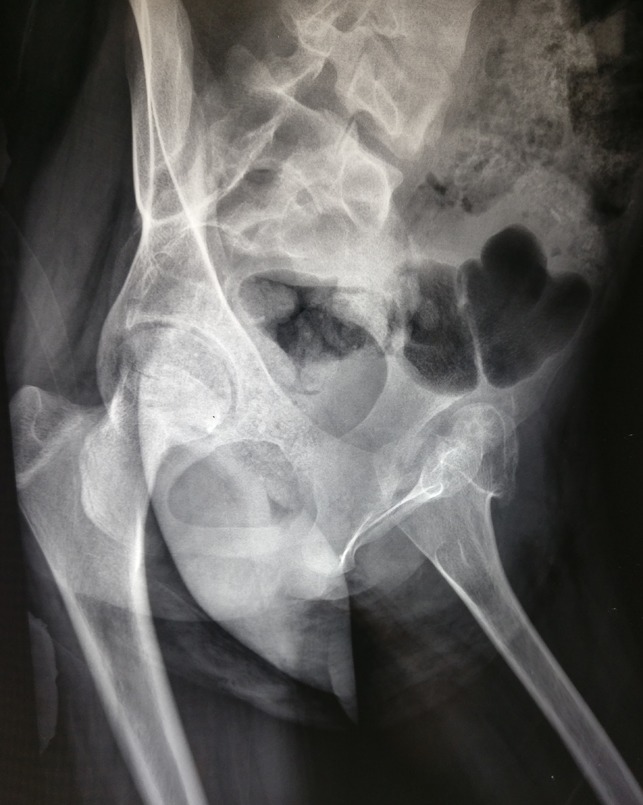
Attempted lateral radiograph demonstrating a deformed femoral head and pseudoacetabulum articulation.

**Figure 3:**
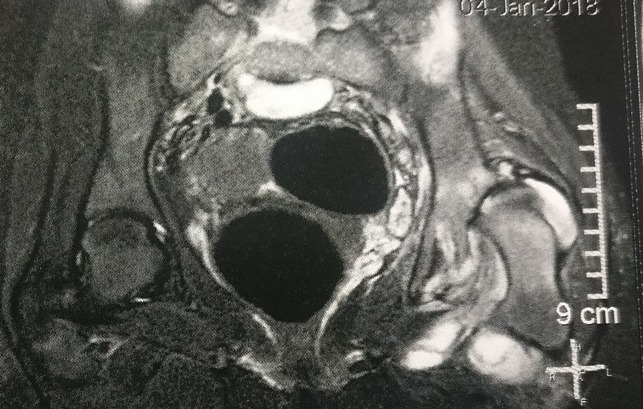
Radiograph demonstrating the coronal T2 fat saturation dysplastic head with adjacent hyperintensity in the soft tissues and localized fluid collection.

**Figure 4:**
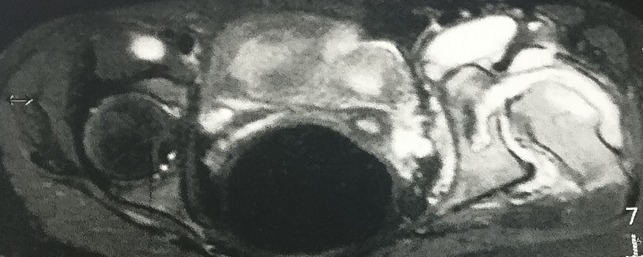
Radiograph demonstrating the axial T2 fat saturation anterior fluid collection.

**Figure 5:**
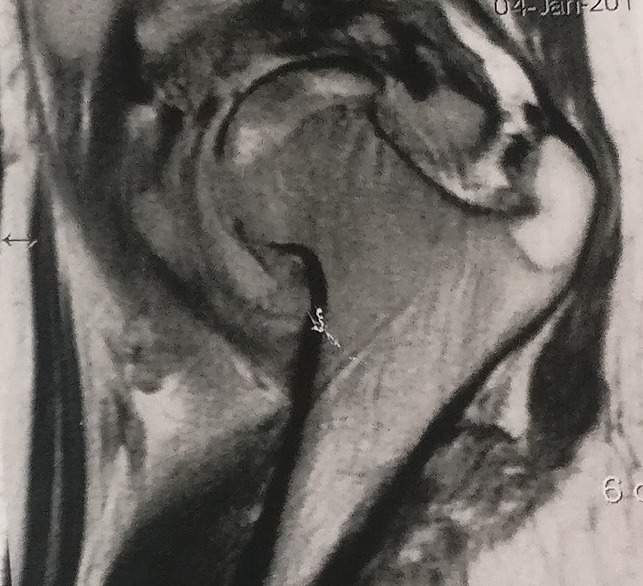
Radiograph showing the PDW T1 Sagittal corroborating the findings in other sections.

**Figure 6:**
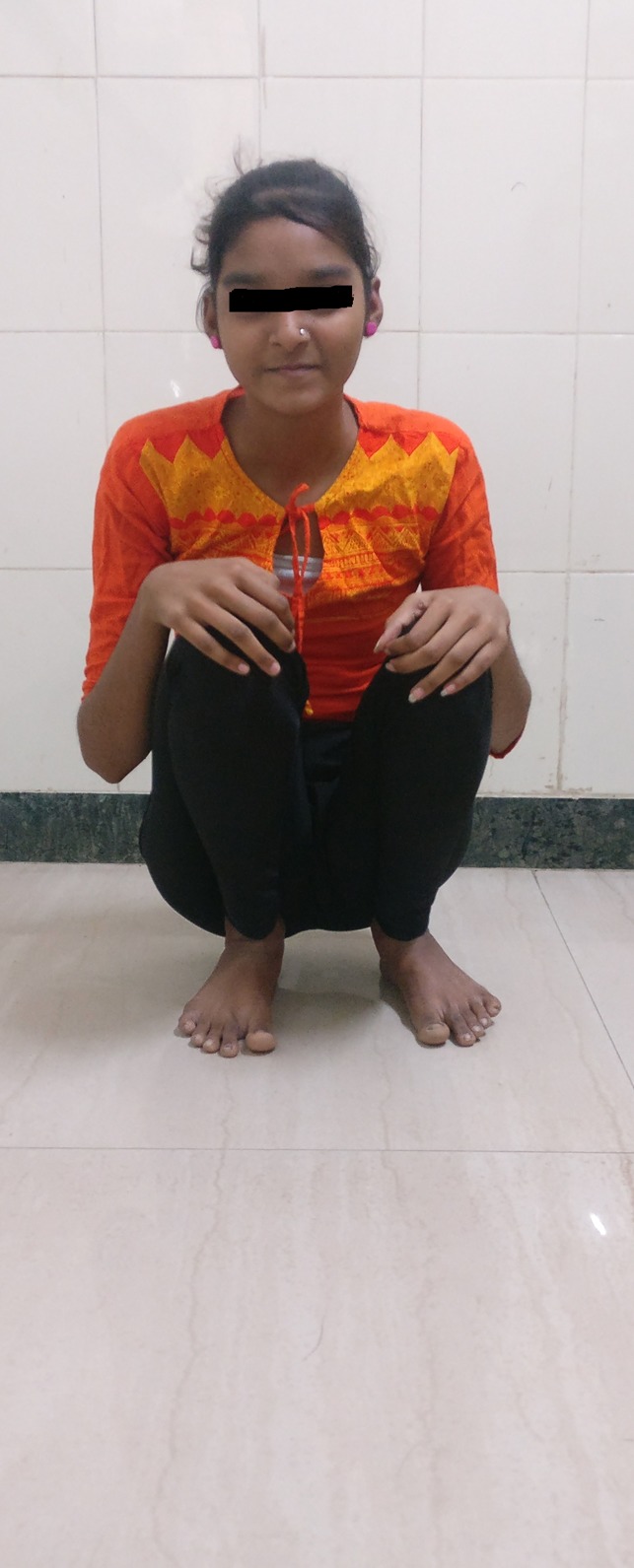
Photograph of the patient at the 1-year follow-up demonstrating good function and ability to squat.

A 14-year-old postmenarchal girl presented to us with an insidious onset left hip pain for a 3-month duration which was gradually increasing. She had an intermittent evening fever which used to resolve either spontaneously or with medications. She also reported of loss of appetite over the past 2 months. The patient's mother informed us that the patient used to lurch to the left side since she started walking around 1.5 years of age. The lurch was painless and nonprogressive. The patient was born at full term by a normal vaginal delivery, and there was no history of any hospitalizations during infancy or childhood. There was no history of developmental delay, fever with chills in infancy, significant trauma, or any intervention on the left hip. She was able to squat and sit cross legged before the onset of left hip pain. Later, her activities were severely restricted, and she had difficulty in carrying out daily activities including squatting and sitting cross legged. On examination, the patient had an antalgic gait. There was fullness and tenderness in Scarpa's triangle and in the gluteal region. Wasting was noted in the gluteal region and thigh. The greater trochanter was displaced proximally, and the swelling in the gluteal region moved on attempted rotation of the thigh. There was a flexion deformity of 30° at the left hip with further flexion possible up to 110° and was painful throughout. Abduction was possible up to 10° and was painful. Adduction was not possible. External rotation was possible up to 10° and was painful, and internal rotation was not possible. An attempted telescoping manoeuvre was painful and was hence not completed. There was a true supratrochanteric shortening of the left lower limb by 4.5 cm. Serological investigations revealed mild normocytic normochromic anemia and a lymphocytic leukocytosis. Erythrocyte sedimentation rate was 55 mm/hr, and quantitative C reactive protein was 46.8 mg/L. Plain radiograph of the left hip revealed a dislocated and deformed femoral head with disruption of the Shenton's arc. The acetabulum was dysplastic with an increased inclination and a widened acetabular teardrop (Figure 1). There was a formation of a secondary “pseudoacetabulum” in the ilium which appeared to articulate with the femoral head (Figure 2). Noncontrast MRI of the hip was performed to ascertain the cause of pain (Figures 3 and 4). There was a fluid collection around the left hip and around the muscles of the left hip region with an anterior outpouching (Figure 5). Surgery was planned for a diagnostic biopsy and to excise the proliferative, diseased, and necrotic tissues. An arthrotomy of the left hip was performed by the Smith-Peterson approach. The abscess was drained and sent for routine microbiological examination. In addition, the cartridge-based nucleic acid amplification test (Gene Xpert) for *Mycobacterium* and liquid broth culture and antibiotic sensitivity was also carried out. The results came back positive for TB with sensitivity to all first-line antitubercular drugs. Pulmonary TB was ruled out by a chest radiograph and sputum microscopic examination and culture in both solid and liquid media. We do not perform QuantiFERON-TB Gold in tube for our patients owing to its questionable role in high endemicity countries and high rates of false positives. A skin tuberculin testing was not performed because of the history of Bacillus Calmette-Guerin (BCG) vaccination. The patient was started on weight-based first-line antitubercular chemotherapy with isoniazid, rifampicin, pyrazinamide and ethambutol, and hematinics for a period of 3 months. She showed both clinical and serological signs of recovery, gradually over the next 3 months. This chemotherapy was later converted to a two-drug regime with weight-based isoniazid and rifampicin for a period of 7 months titrated with serological and clinical recovery as per the institutional protocol. The antitubercular chemotherapy was stopped after a period of 10 months when the patient had resolution of pain. The lurch persisted, and the patient was counseled regarding the need for a replacement options for the dysplastic hip. The patient was concerned regarding the ability to squat and sit cross legged. At the 1-year follow-up, the patient resumed all her activities including squatting and sitting cross legged and remained painless with residual lurch and limb shortening on the left side (Figure 6).

## Discussion

Developmental dysplasia of hip encompasses a wide spectrum of anatomic hip abnormalities that affect the development and stability of the hip. These abnormalities can range from subtle acetabular dysplasia to complete subluxation or dislocation of the femoral head. Developing countries do offer the other end of spectrum of this disease for a dislocated hip, many presenting after the walking age. Presentation in the periadolescent age group is also not infrequently found. The aim of treatment usually in cases presenting early is to achieve a stable and concentrically-located joint and satisfactory development of the hip which is achieved surgically. It is assumed that the outcome of treatment is inversely related to the age at presentation. Left untreated, these dysplastic hips lead to an abnormal gait pattern and osteoarthritis by early adulthood.^1^ Surgical intervention at old age is met with other sets of difficulties like extensive soft-tissue contractures, high riding femoral head, and a dysplastic acetabulum added to the risk of complications including osteonecrosis of femoral head and uncertainty about the radiological and clinical outcome of the procedure. Many of these hips even if left untreated function well, especially bilateral cases, for another 2 to 3 decades before developing changes of osteoarthritis. Few authors suggest to avoid surgical intervention beyond the age of 8 to 10 years and to follow the policy of wilful neglect and continued observation owing to the poor remodeling potential of the acetabulum.^2^ However, there are others who suggest treatment by open reduction adequate shortening (up to 5 cm) with derotation, and limited varization if needed, tight capsulorrhaphy, and appropriate pelvic reconstruction (Salter or triple acetabular osteotomy) with an average age at operation around 10.6 years (range 8 to 18 years) reporting good results in 79% cases.^3^ They offer that older children presenting with unilateral or bilateral dislocation of the hip should be treated, unless there is an underlying systemic disorder contraindicating the operation. Addition of TB to this already existing controversy further complicates the matter and limits the treatment options to few. The pain, loss of movement, and progressive development of deformity results in the loss of function of an already compromised hip with a poor overall function. Early diagnosis is of paramount importance in the treatment of TB and its outcome. In the past 3 decades, extensive research was performed on the diagnosis and management of TB.^4^ Besides clinical features, serological, radiological and molecular diagnostic methods including polymerase chain reaction are available easily. Osteoarticular TB still comprises a significant number of patients among the total TB burden in our country. The cases of multidrug-resistant TB is also on a rise secondary to inappropriate antitubercular chemotherapy without drug testing. Synovial TB is primarily considered to be a medical disease and surgical intervention is reserved for advanced joint destruction. In this case, surgical options regarding reconstruction of the hip were not available owing to a previously dysplastic state, and hence, more stress was given to the medical mode of treatment. The morphological classification proposed by Shanmugasundaram^5^ for tuberculous hips cannot be effectively used in this case because the dislocation is not secondary to TB but an independent preexisting anomaly. Early institution of appropriate chemotherapy is of paramount importance in the management of joint TB to prevent irreversible destruction and deformities. The patient was counseled regarding the future possible courses of the current state and development of osteoarthritis that may require surgical intervention in the form of complex arthroplasty or arthrodesis in single or multiple settings. The parents were also counseled regarding the guarded outcomes of arthroplasty in these cases and that one procedure might not be the best in that case.

Double attack at the hip with dysplasia and TB poses double set of problems, both for the patient and the treating surgeon. The primary aim should be targeted toward an early diagnosis of the infection, early institution of medical management, and uneventful resolution of the disease so as to save adequate anatomy for future reconstruction of the hip.^5,6^ Until that time adequate counseling, reassurance and periodic monitoring are all that is required to be performed. To our knowledge, this is the first such case being reported in the literature.
